# Association of Coronary Artery Calcium Score with Cardiovascular Outcomes in Patients Without Known Coronary Artery Disease

**DOI:** 10.3390/life16050755

**Published:** 2026-05-01

**Authors:** Snežana Bjelić, Dragana Dabović, Teodora Čekić, Andrej Preveden, Nikola Komazec, Marija Bjelobrk

**Affiliations:** 1 Faculty of Medicine, University of Novi Sad, 21000 Novi Sad, Serbia; snezana.bjelic@mf.uns.ac.rs (S.B.);; 2 Institute of Cardiovascular Diseases Vojvodina, 21204 Sremska Kamenica, Serbia

**Keywords:** coronary artery calcium, coronary artery disease, cardiovascular risk, risk stratification, score, major adverse cardiovascular events

## Abstract

Elevated coronary artery calcium (CAC) is associated with increased cardiovascular and all-cause mortality. Coronary artery calcium score (CACS) may aid cardiovascular risk assessment beyond traditional models, particularly in primary prevention populations. This study aimed to evaluate the association between CACS and major adverse cardiovascular events (MACE) and its relationship with conventional cardiovascular risk models in patients without established coronary artery disease. We conducted a retrospective analysis of patients aged >40 years with at least one cardiovascular risk factor who underwent computed tomography coronary angiography. Data on cardiovascular risk factors, medication use, atherosclerotic cardiovascular disease (ASCVD) score, MESA score, and CACS were collected. Among 100 patients (61% male; mean age 57.1 ± 10.7 years), 47% were at intermediate ASCVD risk. Median CACS was 65.0 (IQR 0.0–383.3). CACS was significantly associated with ASCVD and MESA scores. In multivariable analysis, glucose level, ASCVD score, and MESA score were significantly associated with MACE, while CACS showed a borderline association. These findings should be interpreted as exploratory and suggest that CACS reflects overall cardiovascular risk burden and may contribute to risk assessment when integrated with conventional approaches. Further prospective studies are needed to validate these findings.

## 1. Introduction

Coronary artery disease (CAD) is often asymptomatic until a major event occurs: approximately 50% to two-thirds of patients experience sudden cardiac death or nonfatal myocardial infarction as the first manifestation of disease [1,2]. Therefore, identifying individuals with subclinical CAD who may benefit from intensive primary prevention remains a major clinical challenge.

Risk assessment of atherosclerotic cardiovascular disease (ASCVD) has traditionally relied on models derived from the Framingham Heart Study, including the Framingham Risk Score (FRS). However, as FRS was developed in a predominantly White, middle-aged population from a single U.S. community, its generalizability is limited [3]. Although several alternative risk models have been proposed, traditional approaches account for only 65–80% of future cardiovascular events [4].

Coronary artery calcium (CAC), quantified by the coronary artery calcium score (CACS), reflects the overall burden of atherosclerotic plaque. Large prospective studies have shown that elevated CAC is associated with increased cardiovascular and all-cause mortality. Moreover, incorporating CACS into risk prediction models improves the discriminative ability of traditional scores, including ASCVD and FRS [5].

The aim of this study was to evaluate the association between CACS and major adverse cardiovascular events (MACE) and to examine its relationship with conventional cardiovascular risk models in patients without established coronary artery disease (CAD).

## 2. Materials and Methods

We conducted a retrospective, non-randomized pilot study at the Institute of Cardiovascular Diseases Vojvodina between 2019 and 2022. A total of 100 patients of both sexes, aged >40 years, with at least one cardiovascular risk factor were included. All participants underwent multislice computed tomography (MSCT) coronary angiography and were followed for up to 3 years. Written informed consent was obtained from all participants, and the study protocol was approved by the local Ethics Committee.

Patients with previously documented significant coronary artery disease (CAD) confirmed by MSCT or invasive coronary angiography, prior revascularization (coronary artery bypass grafting (CABG) and/or percutaneous coronary intervention (PCI)), or those who declined participation were excluded.

### 2.1. Clinical Assessment and Risk Factors

Baseline assessment included age, sex, blood pressure, glucose levels, lipid profile (total cholesterol, LDL-C, HDL-C), body mass index (BMI), smoking status, family history of CAD, and use of antihypertensive, lipid-lowering, and antiplatelet therapies. Serum creatinine was measured, and estimated glomerular filtration rate (eGFR) was calculated using the CKD-EPI equation [6].

### 2.2. Risk Scores

The following validated risk scores were calculated:

• Atherosclerotic cardiovascular disease (ASCVD) risk score [7].
• Multi-Ethnic Study of Atherosclerosis (MESA) 10-year coronary heart disease (CHD) risk score with CAC [8].
• Coronary artery calcium score (CACS), derived from MSCT.

### 2.3. CAC Assessment

Non-contrast, electrocardiogram-gated cardiac CT scans were performed using either electron-beam tomography or multidetector CT. CAC was quantified using the Agatston scoring method [9]. Patients were stratified into four groups: CACS 0, 1–99, 100–399, and ≥400, corresponding to increasing coronary artery calcium burden.

### 2.4. Follow-Up and Outcomes

Patients were followed for a minimum of 6 months and up to 3 years for the occurrence of major adverse cardiovascular events (MACE), defined as acute myocardial infarction (AMI), acute ischemic stroke (AIS), hospitalization for cardiovascular causes, or all-cause mortality.

To improve interpretability, cardiovascular hospitalizations were defined as admissions due to documented cardiovascular causes (e.g., unstable angina, heart failure, arrhythmia), and their contribution to the composite endpoint was analyzed separately. Hard cardiovascular events were defined as myocardial infarction, acute ischemic stroke, or death, and were analyzed descriptively due to their low frequency.

### 2.5. Statistical Analysis

Statistical analysis was performed using SPSS version 17.0. Continuous variables were expressed as mean ± standard deviation or median (interquartile range, IQR), as appropriate, and categorical variables as frequencies and percentages. Group comparisons were performed using the independent samples *t*-test or Mann–Whitney U test for continuous variables and the chi-square or Fisher’s exact test for categorical variables. Model performance was assessed using receiver operating characteristic (ROC) curve analysis, and discrimination was expressed as the area under the curve (AUC). Logistic regression analysis was used to identify predictors of MACE.

Given the skewed distribution of CACS values, analyses were performed using both categorical and regression-based approaches. Due to the limited sample size and number of events, time-to-event analysis (e.g., Cox proportional hazards modeling) and advanced measures of incremental predictive value (e.g., NRI and IDI) were not applied.

A two-sided *p*-value < 0.05 was considered statistically significant.

## 3. Results

### 3.1. Baseline Characteristics of the Study Population

Among the 100 included patients, 61% were male, and the mean age was 57.1 ± 10.7 years. Nearly half of the cohort (47%) had an intermediate ASCVD risk. The median coronary artery calcium score (CACS) was 65.0 (interquartile range [IQR] 0.0–383.3). During the follow-up period, 29% of patients experienced major adverse cardiovascular events (MACE), with hospitalization being the most frequent event (24%). Detailed clinical characteristics, cardiovascular risk factors, and mean values of the MESA score and CACS are presented in Table 1 and Table 2.

### 3.2. Predictors of Major Adverse Cardiovascular Events

Further, we investigated the influence of examined parameters on cardiovascular outcomes (MACE—Major Adverse Cardiovascular Events). An association was observed between MACE and ASCVD score (*p* = 0.037), as well as CACS (*p* = 0.067), which is shown in Table 3.

The proportion of MACE increased across ASCVD risk categories: 15.4% in the low-risk group, 9.1% in the borderline group, 34% in the intermediate-risk group, and 50% in the high-risk group.

When analyzed across standard CACS categories, a trend toward a higher incidence of MACE was observed with increasing calcium burden. The proportion of events was 19.4% in patients with CACS = 0, 30.8% in the 1–99 group, 19.0% in the 100–399 group, and 50.0% in those with CACS ≥ 400.

Although a higher event rate was observed in patients with CACS ≥ 400, this association did not reach statistical significance (*p* = 0.067).

Notably, patients with very high coronary artery calcium scores (CACS > 1000) exhibited the highest incidence of MACE, with events occurring in 70% of this subgroup.

No statistically significant associations were observed for the remaining variables. Univariate logistic regression analysis identified glycemia, ASCVD score, MESA score, and CACS as predictors of MACE (Table 4). In multivariable analysis, CACS showed a borderline association with MACE (OR 1.47, 95% CI 0.997–2.176, *p* = 0.052).

Although this did not reach conventional statistical significance, the observed trend suggests a potential relationship between increasing coronary artery calcium burden and adverse cardiovascular outcomes.

A progressive shift toward higher ASCVD risk categories was observed with increasing CACS values. Among patients with CACS = 0, the majority were classified as low risk (51.6%), with only 6.5% in the high-risk category.

In contrast, patients with higher CACS demonstrated a marked redistribution toward intermediate and high-risk groups. In the CACS 100–399 group, 57.1% of patients were classified as intermediate risk and 23.8% as high risk. This trend was even more pronounced in patients with CACS ≥ 400, where the majority were categorized as intermediate (59.1%) or high risk (36.4%), and no patients were classified as low risk.

Overall, a statistically significant association was observed between CACS and ASCVD risk categories (*p* < 0.0005), indicating that increasing coronary artery calcium burden is strongly related to higher estimated cardiovascular risk (Table 5).

A significant increase in MESA score values was observed across CACS categories. Median MESA score rose progressively from 3.40 in patients with CACS = 0 to 21.35 in those with CACS ≥ 400.

This trend was consistent across percentiles, indicating a marked shift toward higher estimated cardiovascular risk with increasing coronary artery calcium burden.

Overall, a strong and statistically significant association was observed between CACS and MESA score (*p* < 0.0005) (Table 6).

**Figure 1:**
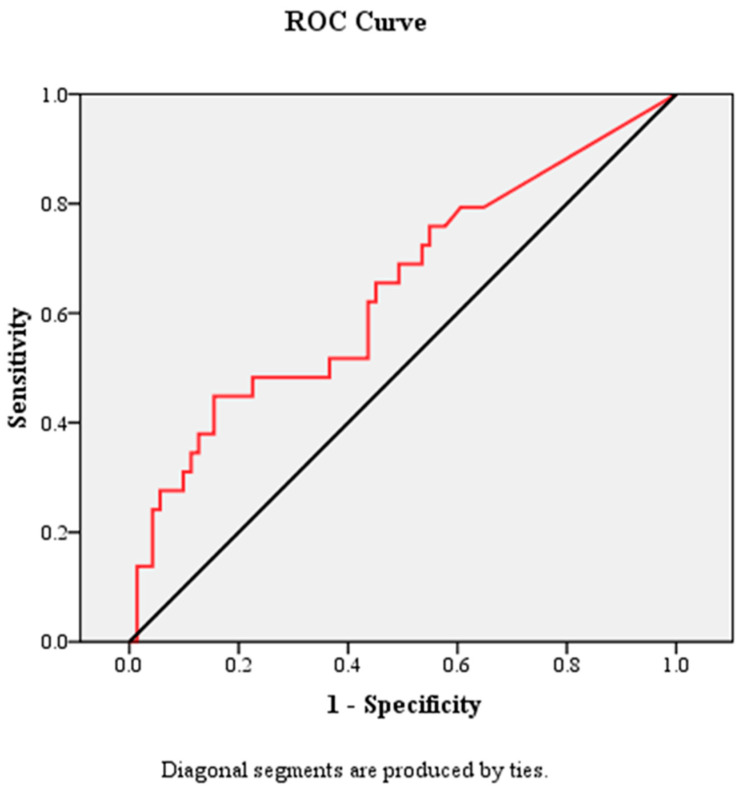
Discriminatory performance of CACS for prediction of major adverse cardiovascular events: ROC curve analysis.

Receiver operating characteristic (ROC) curve analysis demonstrated that CACS had modest but statistically significant discriminatory ability for predicting MACE, with an area under the curve (AUC) of 0.643 (95% CI 0.518–0.768, *p* = 0.025) (Figure 1).

The optimal cut-off value was 359.5 Agatston units, which corresponds approximately to the clinically relevant threshold of 400, yielding a sensitivity of 44.8% and specificity of 81.7%.

The composite endpoint was largely driven by cardiovascular hospitalizations, which should be taken into account when interpreting regression and ROC analyses.

## 4. Discussion

Current guidelines recommend global risk assessment as the first step in evaluating atherosclerotic cardiovascular disease (ASCVD) [7,10]. In individuals at borderline or intermediate risk, further refinement requires the inclusion of “risk-enhancing” factors, including family history, metabolic abnormalities, inflammatory biomarkers, and imaging of subclinical atherosclerosis. Among available imaging modalities, coronary artery calcium (CAC) scoring is particularly valuable and is endorsed by multiple guidelines to improve risk stratification and guide preventive strategies, including pharmacological therapy and lifestyle modification [10].

The present study was designed as a pilot, real-world analysis to evaluate the consistency of CACS performance in relation to established risk scores (ASCVD and MESA) in individuals without known coronary artery disease and with at least one cardiovascular risk factor, reflecting a real-world primary prevention population.

Numerous studies have demonstrated that CAC score is associated with major adverse cardiovascular events (MACE), beyond traditional risk factors such as age, cholesterol levels, hypertension, smoking, and diabetes [5,11,12,13,14]. Its principal clinical utility lies in reclassifying individuals with intermediate ASCVD risk into higher or lower risk categories, thereby refining therapeutic decision-making.

In our cohort, 47% of patients were classified as intermediate risk, highlighting the clinical relevance of this subgroup. Event rates increased across CACS categories, ranging from 20% in patients with CACS = 0 to 70% in those with CACS > 1000; however, this subgroup was small and should be interpreted with caution.

The concept of “vascular age” has emerged as a complementary measure to chronological age in cardiovascular risk assessment. While traditionally derived from risk scores such as FRS and SCORE, CAC provides a direct estimate of cumulative atherosclerotic burden and may better reflect biological vascular aging [14,15,16,17]. In our study, despite a mean chronological age of 57 years, the wide distribution of CACS values suggests substantial variability in vascular aging among individuals.

Hypertension and dyslipidemia were the most prevalent cardiovascular risk factors in our cohort. Prior studies have confirmed strong associations between systolic blood pressure, dyslipidemia subtypes, and CAC extent [16,17,18,19]. However, these variables did not independently predict MACE after adjustment for CACS, suggesting that CAC may integrate the cumulative impact of multiple risk exposures and serve as a more comprehensive marker of cardiovascular risk.

Consistent with previous studies [20,21,22], ASCVD score was significantly associated with cardiovascular outcomes, whereas CACS showed a trend toward increased risk. The composite endpoint was predominantly driven by cardiovascular hospitalizations, with a low number of hard events, including only one myocardial infarction, no cardiovascular deaths, and a small number of ischemic strokes. Notably, the majority of hospitalizations were related to atrial fibrillation and optimization of medical therapy, which may influence the interpretation of clinical outcomes. This predominance may partly explain the modest discriminatory performance of CACS observed in our study.

We also observed a statistically significant association between CACS and MESA score, supporting consistency between imaging-based and traditional risk assessment approaches. Event rates increased progressively across ASCVD categories and CACS strata. Importantly, multivariable analysis showed only a borderline association between CACS and MACE (OR 1.47, 95% CI 0.997–2.176, *p* = 0.052), rather than a definitive independent predictive effect. The potential influence of medical therapy (e.g., statins and antihypertensive treatment) should also be considered, as it may confound observed associations.

Receiver operating characteristic (ROC) analysis demonstrated modest but statistically significant discriminatory ability of CACS (AUC 0.643, *p* = 0.025), with a cut-off value approximating 400 Agatston units. Given the limited sample size, more advanced statistical approaches were not applied.

Taken together, these findings suggest a potential role of CACS in risk assessment, particularly in individuals at intermediate risk. These results should, however, be interpreted with caution given the modest discriminatory performance and borderline multivariable findings.

Further large-scale, prospective, multicenter studies are warranted to validate these findings and better define the role of CACS in contemporary risk models. Future research should explore integration with multimodal data, including advanced imaging biomarkers, inflammatory markers, and genetic risk scores. Structural and anatomical parameters (e.g., chest wall geometry, modified Haller index) may also contribute to improved risk evaluation [23].

Machine learning and artificial intelligence approaches may further enhance the clinical utility of CACS in personalized medicine.

### Limitations

Several limitations should be acknowledged. First, this was a single-center, retrospective pilot study with a relatively small sample size, which may limit statistical power and generalizability. Second, the follow-up duration was modest (up to 3 years) compared with larger prospective CAC studies. Third, the composite endpoint was predominantly driven by cardiovascular hospitalizations, with a low number of hard events, which may influence clinical interpretation.

Fourth, the inclusion of patients already receiving preventive therapies may have attenuated the predictive value of conventional risk factors. Additionally, more advanced statistical analyses were not feasible due to the limited number of events.

Finally, as CACS quantifies only calcified plaque, it may underestimate cardiovascular risk in younger individuals or in those with predominantly non-calcified atherosclerotic lesions.

## 5. Conclusions

Coronary artery calcium score (CACS), together with MESA score, ASCVD score, glucose level, and age, was associated with cardiovascular outcomes in patients without established coronary artery disease. These results should be interpreted as exploratory and suggest a potential role of CACS in risk refinement within a real-world primary prevention setting. Further large-scale prospective studies are needed to validate these findings and to better define the role of CACS in cardiovascular risk assessment.

## Figures and Tables

**Table 1:** Baseline characteristics and clinical profile of the study population.

Parameter	*n* (%)
Demographics and risk factors	
Male sex	61 (61%)
Diabetes mellitus type 2 (DMT2)	15 (15%)
Arterial hypertension (HTA)	74 (74%)
Hyperlipidemia (HLP)	62 (62%)
Current smoking	44 (44%)
Obesity (BMI ≥ 30 kg/m^2^)	29 (29%)
Sedentary lifestyle	52 (52%)
Family history of CAD	50 (50%)
Medical therapy	
Antihypertensive therapy	70 (70%)
Statin therapy	61 (61%)
Antiplatelet therapy	75 (75%)
ASCVD risk categories	
Low (<5%)	26 (26%)
Borderline (5–7%)	11 (11%)
Intermediate (7.5–20%)	47 (47%)
High (>20%)	16 (16%)
Coronary artery calcium score (CACS)	
0	31 (31%)
1–99	26 (26%)
100–399	21 (21%)
≥400	22 (22%)
Major adverse cardiovascular events (MACE)	
Acute myocardial infarction	1 (1%)
Ischemic stroke	4 (4%)
Death	0 (0%)
Cardiovascular hospitalization	24 (24%)
Total MACE	29 (29%)

Abbreviations: DMT2—diabetes mellitus type 2; HTA—arterial hypertension; HLP—hyperlipidemia; ASCVD—atherosclerotic cardiovascular disease; CACS—coronary artery calcium score; MACE—major adverse cardiovascular events.

**Table 2:** Distribution of continuous cardiovascular risk factors and risk scores.

Parameter		Mean ± SD (N = 100)			Min	Max
Age (years)		57.1 ± 10.7			40.0	76.0
Glucose (mmol/L)		6.2 ± 1.6			4.2	15.2
SBP (mmHg)		128.9 ± 19.4			80.0	200.0
DBP (mmHg)		79.7 ± 15.3			50.0	130.0
Total cholesterol (mmol/L)		5.1 ± 1.4			2.7	9.4
LDL-C (mmol/L)		3.1 ± 1.1			1.3	7.1
HDL-C (mmol/L)		1.2 ± 0.4			0.4	2.8
Triglycerides (mmol/L)		1.5 ± 0.7			0.6	4.3
BMI (kg/m^2^)		28.2 ± 5.1			19.7	44.3
Creatinine (µmol/L)		90.0 ± 29.1			40.0	245.0
eGFR (mL/min/1.73 m^2^)		81.6 ± 20.0			19.2	117.5
Distribution of selected scores (percentiles)						
Parameter	25th Percentile		Median	75th Percentile		
MESA score (N = 84)	4.6		13.0	19.4		
CACS	0.0		65.0	383.3		

Abbreviations: SBP—systolic blood pressure; DBP—diastolic blood pressure; LDL-C—low-density lipoprotein cholesterol; HDL-C—high-density lipoprotein cholesterol; BMI—body mass index; eGFR—estimated glomerular filtration rate; CACS—coronary artery calcium score; MESA—Multi-Ethnic Study of Atherosclerosis.

**Table 3:** Association between clinical variables, risk scores, and major adverse cardiovascular events (MACE).

Parameter	MACE		*p* Value
	No (%)	Yes (%)	
Gender			
Female	29 (74.4)	10 (25.6)	0.654
Male	42 (68.9)	19 (31.1)	
DMT2			
No	63 (74.1)	22 (25.9)	0.126
Yes	8 (53.3)	7 (46.7)	
HLP			
No	30 (78.9)	8 (21.1)	0.256
Yes	41 (66.1)	21 (33.9)	
HTA			
No	18 (69.2)	8 (30.8)	0.807
Yes	53 (71.6)	21 (28.4)	
Current smoking			
No	39 (69.6)	17 (30.4)	0.826
Yes	32 (72.7)	12 (27.3)	
Obesity			
No	51 (71.8)	20 (28.2)	0.811
Yes	20 (69)	9 (31)	
“Lifestyle”—sedentary			
No	6 (75)	2 (25)	1.000
Yes	36 (69.2)	16 (30.8)	
Family history			
No	34 (68)	16 (32)	0.660
Yes	37 (74)	13 (26)	
Antihypertensive therapy			
No	23 (76.7)	7 (23.3)	0.478
Yes	48 (68.6)	22 (31.4)	
Statin therapy			
No	28 (71.8)	11 (28.2)	1.000
Yes	43 (70.5)	18 (29.5)	
ASCVD Score			
Low < 5%	22 (84.6)	4 (15.4)	0.037
Borderline 5–7%	10 (90.9)	1 (9.1)	
Intermediate 7.5–20%	31 (66)	16 (34)	
High > 20%	8 (50)	8 (50)	
CACS			
CACS 0	25 (80.6)	6 (19.4)	0.067
CACS 1–99	18 (69.2)	8 (30.8)	
CACS 100–399	17 (81)	4 (19)	
CACS ≥ 400	11 (50)	11 (50)	

Abbreviations: DMT2—diabetes mellitus type 2; HTA—arterial hypertension; HLP—hyperlipidemia; ASCVD score—atherosclerotic cardiovascular disease score; CACS—coronary artery calcium score; MACE—major adverse cardiovascular events.

**Table 4:** Univariate logistic regression analysis for predictors of major adverse cardiovascular events (MACE).

Parameter	OR (95% CI)	*p* Value
Gender	1.312 (0.533–3.227)	0.554
Age	1.045 (0.999–1.092)	0.054
DMT2	2.506 (0.814–7.714)	0.109
Glycemia	1.330 (1.003–1.762)	0.047
HLP	1.921 (0.750–4.920)	0.174
T-HOL	1.091 (0.797–1.494)	0.586
LDL	1.241 (0.838–1.838)	0.281
HDL	0.904 (0.286–2.859)	0.863
TGL	0.696 (0.352–1.375)	0.297
HTA	0.892 (0.337–2.362)	0.817
SBP	0.984 (0.961–1.008)	0.181
DBP	0.991 (0.963–1.021)	0.564
Smoking	0.860 (0.359–2.063)	0.736
Obesity	1.147 (0.448–2.942)	0.775
BMI	1.010 (0.929–1.098)	0.818
“Lifestyle”—sedentary	1.333 (0.242–7.337)	0.741
Family history	0.747 (0.314–1.778)	0.509
Antihypertensive therapy	1.506 (0.562–4.033)	0.415
Statin therapy	1.066 (0.438–2.590)	0.889
Creatinine	0.998 (0.982–1.013)	0.774
eGFR	0.995 (0.974–1.017)	0.642
ASCVD score	1.881 (1.161–3.048)	0.010
MESA score	1.055 (1.004–1.109)	0.033
Ca Score	1.001 (1.000–1.002)	0.023
CASC	1.473 (0.997–2.176)	0.052

Abbreviations: DMT2—diabetes mellitus type 2; HLP—hyperlipidemia; T-HOL—total cholesterol level (mmol/L); LDL—low density lipoprotein level (mmol/L); HDL—high density lipoprotein level (mmol/L); TGL—triglyceride level (mmol/L); HTA—arterial hypertension; SBP—systolic blood pressure (mmHg); DBP—diastolic blood pressure (mmHg); BMI—body mass index (kg/m^2^); eGFR estimated glomerular filtration rate (ml/min/1.73 m^2^); ASCVD score—atherosclerotic cardiovascular disease score; CACS—coronary artery calcium score; MACE—major adverse cardiovascular events.

**Table 5:** Distribution of ASCVD risk categories across coronary artery calcium score (CACS) groups.

CACS Category		ASCVD Score			
	Low < 5%	Borderline 5–7%	Intermediate 7.5–20%	High > 20%	Total
0	16 (51.6%)	3 (9.7%)	10 (32.3%)	2 (6.5%)	31 (100%)
1–99	8 (30.8%)	5 (19.2%)	12 (46.2%)	1 (3.8%)	26 (100%)
100–399	2 (9.5%)	2 (9.5%)	12 (57.1%)	5 (23.8%)	21 (100%)
≥400	0 (0.0%)	1 (4.5%)	13 (59.1%)	8 (36.4%)	22 (100%)
Total	26 (26.0%)	11 (11.0%)	47 (47.0%)	16 (16.0%)	100 (100%)

Abbreviations: CACS—coronary artery calcium score; ASCVD—atherosclerotic cardiovascular disease.

**Table 6:** Distribution of MESA score across coronary artery calcium score (CACS) categories.

CACS Category	25th Percentile	Median	75th Percentile	*p*-Value
0	2.60	3.40	4.40	<0.0005
1–99	5.60	7.05	12.50	
100–399	13.70	15.60	20.40	
≥400	16.30	21.35	28.40	

Abbreviations: CACS—coronary artery calcium score; MESA—Multi-Ethnic Study of Atherosclerosis risk score.

## Data Availability

The original contributions presented in this study are included in the article. Further inquiries can be directed to the corresponding author.

## Abbreviations

The following abbreviations are used in this manuscript:

DMT2	Diabetes mellitus type 2
HTA	Arterial hypertension
HLP	Hyperlipidemia
SBP	Systolic blood pressure
DBP	Diastolic blood pressure
T-CHOL	total cholesterol level
LDL-C	low density lipoprotein level
HDL-C	high density lipoprotein level
TGL	triglyceride level
BMI	body mass index
eGFR	estimated glomerular filtration rate
ASCVD score	Atherosclerotic cardiovascular disease risk score
CACS	Coronary artery calcium score
MACE	Major adverse cardiovascular events
MESA score	Multi-Ethnic Study of Atherosclerosis Risk Score
